# Does the national dental scaling policy reduce inequalities in dental scaling usage? A population-based quasi-experimental study

**DOI:** 10.1186/s12903-019-0881-7

**Published:** 2019-08-14

**Authors:** Eun-Soo Kim, Baek-Il Kim, Hoi In Jung

**Affiliations:** 1 Department of Preventive Dentistry & Public Oral Health, Yonsei University College of Dentistry, 50-1 Yonsei-ro, Seodaemun-gu, Seoul, 03722 Republic of Korea; 2 BK21 PLUS Project, Yonsei University College of Dentistry, 50-1 Yonsei-ro, Seodaemun-gu, Seoul, 03722 Republic of Korea

**Keywords:** Dental scaling, Health care utilization, Inequalities, Universal health coverage, Individual agency

## Abstract

**Background:**

In 2013, the national dental scaling insurance policy was introduced in South Korea. The purpose of this study is to determine the impact of the policy on inequalities in dental scaling usage.

**Methods:**

Data of a nationally representative sample of 1,517,097 people over the age of 20 were obtained from the 2010–2016 Community Health Survey. Respondents who reported that they had not received dental scaling in the past year were defined as dental scaling non-users. The excess prevalence and relative prevalence ratio of dental scaling non-users were calculated for the pre-policy (2010–2012) and post-policy periods (2014–2016) using monthly household income levels. Additionally, trends of dental scaling inequalities were shown as concentration indexes.

**Results:**

The prevalence of dental scaling non-users declined from 58.0 to 48.7% in the highest income group and from 86.3 to 78.8% in the lowest income group. However, the adjusted excess prevalence for the lowest income group compared with the highest had increased from 11.9 (95% CI: 11.9–11.9) to 15.5 (95% CI: 15.5–15.5)%, and the adjusted prevalence ratio increased from 1.19 (95% CI: 1.19–1.20) to 1.29 (95% CI: 1.29–1.30). Absolute and relative concentration indexes of dental scaling non-users increased after policy implementation.

**Conclusions:**

The national dental scaling insurance policy has increased socioeconomic inequalities in dental scaling usage. Because dental care access generally requires high individual agency, expanded dental coverage may have had limited effects in attenuating inequalities and inadvertently widened the gap. To reduce dental care inequalities, universal access with universal dental coverage should be considered.

**Electronic supplementary material:**

The online version of this article (10.1186/s12903-019-0881-7) contains supplementary material, which is available to authorized users.

## Background

Inequality in health is a worldwide phenomenon. These inequalities not only present important health issues but are also considered unfair, unjust, and avoidable social problems [1]. Oral health problems affect many people globally, and inequalities in oral health are easily observed. According to the 2010 global burden of disease, oral diseases affect approximately 3.9 billion people, and untreated dental caries and severe periodontitis were the first and ninth most prevalent diseases, respectively [2]. A recent systematic review reported that low socioeconomic status (SES) was significantly associated with untreated dental caries or caries experience [3]. Oral health inequalities were also observed for periodontitis, tooth loss, oral cancer, and oral health behaviors [4–7].

In South Korea, health insurance system was introduced in 1977 and it took only 12 years to cover the entire population. In 2000, the National Health Insurance Services (NHIS) established by integrating 140 different health insurers. The NHIS directs a two-segmented health insurance system, consisting of national health insurance (NHI) as social insurance and a medical aid program (MAP) as public assistance. NHI covers 97% of the population, and the premium is proportional to individual income. Physicians are reimbursed through a fee-for-services arrangement with out-of-pocket patient fees. The medical aid program, which offers almost the same benefits as the NHI, covers the low-income patients who make up the remaining 3% of the population. In the medical aid program, premiums are waived and out-of-pocket fees are minimized. Physicians are reimbursed with almost the same fee schedule as NHI patients [8] (see Additional file 1).

Recently, the NHIS has expanded its benefits to reduce individual financial burdens and enhance social security. With regard to dental health, annual dental scaling was introduced in 2013 for individuals over 20 years old. In 2017, it was amended to include individuals older than 19. Dental scaling is a non-surgical periodontal treatment that removes plaque and calculus from the oral cavity. It is widely applied to prevent periodontitis and as post-operative care in periodontal surgery [9]. Regular dental attendance and preventive management such as dental scaling can effectively prevent tooth decay and periodontitis by removing dental plaque and calculus, which cause the majority of oral diseases [10, 11]. Before 2013, dental scaling was only covered in the case of postoperative care in periodontal surgery. After 2013, everyone over 20 could receive scaling for the prevention and management of gingivitis and periodontitis (see Additional file 1).

However, some public health interventions can worsen health inequalities. The “inverse care law” refers to the tendency for the availability of good medical care to vary inversely with the need for it in the population it serves [12]. Public health intervention that aims to change individuals’ behaviors can widen the health inequality gap because higher SES groups can more easily access health intervention than lower SES groups [13, 14].

Many frameworks and theoretical models have been proposed to explain and help reduce oral health inequalities on an individual, area, and ecological level [15]. Sabbah et al. [16] reported that social determinants of health have similar effects on oral health and general health, while Watts et al. [17] highlighted a common risk factor approach of integrating a social determinants framework [17]. Also, empirical studies are needed which evaluate the effect of health policy to reduce oral health inequality [18, 19]. Some previous researches have investigated the effectiveness of interventions or policies to reduce oral health inequalities in the utilization of dental care, which includes expanding dental coverage to uninsured people [20–22]. However, the NHIS already covers the entire population, and the NHIS dental scaling policy only expanded the single benefit for dental scaling. Therefore, the purpose of this study is to evaluate whether the national dental scaling insurance policy in Korea reduces inequalities in dental scaling usage.

## Methods

### Study design

This study was conducted using a quasi-experimental design from repeated cross-sectional survey data obtained in South Korea.

### Data and study population

Data were extracted from the 2010–2016 Community Health Survey (CHS) [23]. The CHS is a cross-sectional national health survey that assesses health conditions and the effects of health policies annually. It examines the demographic, health, and medical information of non-institutionalized adults aged 19 years and older. The sample design is a complex survey using stratified-cluster sampling based on households, and all members of the household are interviewed on a one-on-one basis.

Because dental scaling insurance was only available for individuals 20 years and older from 2013 to 2016, we only included individuals over 20 in this study. The data were obtained from the Korea Centers for Disease Control and Prevention (KCDC) and analysis guidelines were strictly observed.

### Prevalence of dental scaling non-users

The following question determined dental scaling usage: “Have you received dental scaling (tartar removal) in the last year?” Based on this question, the prevalence of dental scaling non-users for the past year was estimated.

### Socioeconomic position

We stratified socioeconomic level using non-equivalent monthly household income levels. Participants directly recorded their household income before 2013, whereas they selected their household income levels from options on the questionnaire in 2014–2016. Monthly household income was reported in eight income brackets that we divided into four categories: less than 1 million, between 1 and 3 million, between 3 and 5 million, and more than 5 million won (KRW).

### Covariates

Age (20–34, 35–44, 45–54, 55–64, and ≥ 65), sex, residential area (rural or urban), education level (under 6 years, 6–9 years, 10–12 years, or more than 12 years), insurance status (MAP, former MAP, NHI), current smoking status (current smoker, former smoker, or non-smoker), and subjective oral health (very good, good, moderate, bad, or very bad) were included as covariates.

### Statistical analysis

All statistical analyses accounted for the complex survey design using weights, stratification, and clustering variables. As the dental scaling policy was introduced in July 2013, we distinguished the pre-policy (2010–2012) and post-policy (2014–2016) periods.

We calculated crude absolute differences and relative ratios in dental scaling non-user prevalence across all income levels. We used multivariable Poisson regression models to calculate adjusted absolute differences and relative ratios, adjusting for covariates. A quasi-Poisson model was used to estimate robust variance while reflecting the complex survey design.

For the sensitivity analysis, absolute differences and relative ratios in dental scaling non-user prevalence were also calculated using equivalized and per capita household incomes. Equivalized household income is equal to the household income divided by the square root of the number of household members, whereas per capita income indicates that the household income is divided by the number of household members. The median value of each income category was used, and the median value of the open-ended highest income category was estimated based on the Pareto curve assumption [24].

Relative and absolute concentration indexes were calculated to identify annual trends in dental scaling usage inequality. The concentration index calculates the level of inequality within a given socioeconomic status using the area of the concentration curve. The relative concentration index (RCI) only considers the distribution within inequalities, and it ranges from − 1 to 1 (− 1 and 1 indicate that inequality is completely concentrated in the low or high socioeconomic level, respectively). The absolute concentration index (ACI) considers both the amount and the distribution within inequalities, and it ranges from −μ to μ (μ is mean of the estimator; −μ means the total amount of inequality is concentrated at the low socioeconomic level, μ at the highest).

All statistical analyses used R software version 3.4.3 and R Studio version 1.1.423. The “Survey” package was used to calculate the estimates for the complex survey and the “IC2” package was used to calculate the concentration index.

## Results

The sample consisted of 1,517,097 people over the age of 20 for whom valid information on dental scaling and household income were available (pre-policy period, *N* = 628,572; 2013, *N* = 218,261; post-policy period, *N* = 670,264) (see Additional file 2). There were no considerable changes between pre- and post-policy periods except in dental scaling usage. After the policy was introduced, overall dental scaling usage increased from 30.5 to 40.1% (see Additional file 3).

Dental scaling usage was lower in the following groups: 65 years and older, rural residence, monthly household income of less than 1 million KRW, less than six years of education, medical aid program, current smokers, and very bad subjective oral health. There were almost no differences by sex. The prevalence of dental scaling non-users decreased in all income levels, from 58.0 to 48.7% (a 9.3-percentage-point reduction) in the group with household income ≥5 million KRW, while the change in the < 1 million KRW group was from 86.3 to 78.8% (a 7.5-percentage-point reduction) (Table 1).

**Table 1:** Percentage of people who did not receive dental scaling in the previous year stratified by pre- or post-policy periods using the Community Health Survey 2010–12 and 2014–16 (weighted %)

Prevalence of dental scaling non-users	Pre-policy (2010–2012)	Post-policy (2014–2016)	Differences
Age			
20–34	68.4	58.4	−10.0
35–44	65.5	56.6	−8.9
45–54	64.6	54.5	−10.1
55–64	68.4	56.0	−12.4
65 and older	86.4	77.1	−9.3
Sex			
Male	69.6	60.2	− 9.4
Female	69.4	59.6	−9.8
Residential area			
Urban	67.5	57.8	−9.7
Rural	77.9	69.3	−8.6
Monthly household income (in million KRW)			
< 1	86.3	78.8	−7.5
1–3	73.9	63.8	−10.1
3–5	66.9	56.6	−10.3
≥ 5	58.0	48.7	−9.3
Education			
Under 6 years	87.1	81.0	−6.1
6–9 years	75.0	66.0	−9.0
10–12 years	69.9	60.3	−9.6
More than 12 years	62.3	52.8	−9.5
Insurance status			
MAP	85.8	78.8	−7.0
Former MAP	82.6	73.0	−9.6
NHI	69.0	59.3	−9.7
Smoking			
Current smoker	71.5	63.1	−8.4
Former smoker	67.5	58.7	−8.8
Non-smoker	69.2	59.1	−9.9
Subjective oral health			
Very good	63.7	51.2	−12.5
Good	65.3	52.9	−12.4
Moderate	67.6	57.5	−10.1
Bad	73.4	65.9	−7.5
Very bad	83.9	79.8	−4.1

Despite these improvements, socioeconomic inequalities in dental scaling usage increased. Differences between pre- and post-policy periods were the lowest at −7.5% in the lowest income group (Table 1). The crude excess prevalence of household income in the < 1 million KRW group compared to the household income in the ≥5 million KRW group increased from 28.3% (95% CI: 27.8–28.9) to 30.1% (95% CI: 29.6–30.7), and the crude prevalence ratio increased from 1.49 (95% CI: 1.49–1.49) to 1.61 (95% CI: 1.61–1.62). In the multivariable regression, the adjusted excess prevalence of household income in the < 1 million KRW group compared to the household income in the ≥5 million KRW group increased from 11.9 (95% CI: 11.9–11.9) to 15.5 (95% CI: 15.5–15.5)%, and the adjusted prevalence ratio increased from 1.19 (95% CI: 1.19–1.20) to 1.29 (95% CI: 1.29–1.30), respectively (Table 2).

**Table 2:** Relative ratio and absolute differences in prevalence of dental scaling non-users by income before and after the national dental scaling insurance policy, Community Health Survey 2010–12 and 2014–16, South Korea, ages 20 and older

Monthly household Income (million KRW)	Relative ratio (95% CI)		Absolute difference % (95% CI)	
	Pre–policy (2010–2012)	Post–policy (2014–2016)	Pre–policy (2010–2012)	Post–policy (2014–2016)
Crude				
5 ≤	1	1	0	0
3–5	1.15 (1.15–1.16)	1.16 (1.16–1.16)	9.0 (8.4–9.5)	8.0 (7.4–8.5)
1–3	1.27 (1.27–1.28)	1.31 (1.31–1.31)	15.9 (15.4–16.4)	15.1 (14.6–15.6)
< 1	1.49 (1.49–1.49)	1.62 (1.61–1.62)	28.3 (27.8–28.9)	30.1 (29.6–30.7)
*Adjusted				
5 ≤	1	1	0	0
3–5	1.11 (1.11–1.11)	1.12 (1.12–1.13)	6.9 (6.9–6.9)	6.6 (6.6–6.6)
1–3	1.17 (1.16–1.17)	1.20 (1.20–1.20)	10.2 (10.2–10.3)	10.4 (10.3–10.4)
< 1	1.19 (1.19–1.20)	1.29 (1.29–1.30)	11.9 (11.9–11.9)	15.5 (15.5–15.5)

*A multivariable Poisson regression was used with adjustments for age, sex, residence area, education years, insurance status, current smoking, and subjective oral health

The sensitivity analysis using equivalized and per capita household income also showed consistent trends of increased inequalities in the lowest income level. Differences in dental scaling non-users between pre- and post-policy periods were the lowest with −7.0% (equivalized) and −7.2% (per capita) in the 5th income group. The adjusted relative ratio in the 5th income group compared to the 1st income group increased from 1.21 to 1.30 (equivalized) and 1.20 to 1.28 (per capita). The absolute difference compared to the 1st quantile group also increased in the 5th quantile income group (see Additional file 4).

The relative and absolute concentration indices of dental scaling usage increased after the policy implementation. The RCI increased from −0.063 in 2010 to −0.080 in 2016, whereas the ACI increased from −0.044 from −0.047 (Table 3). Increase of the RCI indicates that the distribution of dental scaling non-users was more skewed in the low-income population. However, increase of the ACI was less than that of the RCI, because it was compensated by the decreased total prevalence of dental scaling non-users. These results are in the same context as the other results in Table 2 or Additional file 4, which show that increased inequalities resulted in the lowest income group, even though the dental scaling usage increased.

**Table 3:** Relative and absolute concentration indices in prevalence of dental scaling non-users by monthly household income, Community Health Survey 2010–16, South Korea, ages 20 and older

	Pre-policy				Post-policy		
	2010	2011	2012	2013	2014	2015	2016
Relative concentration index	−0.063	−0.066	−0.065	−0.068	−0.076	−0.081	−0.080
Absolute concentration index	−0.044	−0.046	−0.045	−0.045	−0.047	−0.048	−0.047

## Discussion

We investigated whether the national dental scaling insurance policy in Korea reduced inequalities in dental scaling usage and found that inequalities in dental scaling usage have widened since implementation of the policy, especially in the lowest income group. To understand why their healthcare utilization has become more restricted, we need to consider the social context of the healthcare system and its relation to low-income patients.

Previous studies reported the mixed effect of national dental insurance policies in dental care inequalities. Hernández-Vásquez et al. reported that the Universal Health Assurance in Peru decreased inequalities in use of oral health services [20], and Cornejo-Ovalle et al. reported declined socioeconomic inequalities in the utilization of dental care after the major healthcare reform in Chile [21]. However, there was no reduction in the socioeconomic gap in dental care and preventive health care after the Affordable Care Act Medicaid expansion in the U.S. [22]. Tudor argued that the inverse care law operates more completely where medical care is most exposed to market forces, and it can operate beyond the healthcare system [12, 25]. McLaren et al. argued that agentic intervention targets to change individual health behavior are more likely to worsen social inequalities in health, while structural intervention that targets the broader social conditions in which behaviors occur is less likely to do so [26]. McGill et al. determined that upstream interventions such as taxing, subsidies, and economic incentives were effective in reducing inequalities in healthy diets, but downstream interventions such as individual education or counseling increased inequalities [27]. In these respects, nationwide healthcare reform in the case of Peru and Chile which includes introducing dental coverage to uninsured people was extensive and structural, and thus could largely reduce the operation of market forces in dental care services. However, expanding single benefits in the case of NHIS dental scaling policy still might require high individual agency for patients to visit dental clinics. Downstream interventions focusing on behavior changes also induced inequalities in a number of areas, including cardiovascular disease, diets and obesity, smoking cessation, and breastfeeding [28–31]. To avoid unintended inequalities, sociocultural-environmental conditions in which health behaviors occur should receive greater consideration [32].

People in the lowest income group were less likely to receive dental scaling in both the pre- and post-policy periods. Economic problems could remain regarding insurance status among low-income people for the following reasons. First, there were about 1.4 million households whose NHI benefits were restricted in 2016 because they were more than six payments behind in their insurance payments [33]. Because registration requirements for the MAP are quite strict, many low-income citizens do not qualify for the program and must pay the NHI premium. Second, every NHI beneficiary must pay the same out-of-pocket costs regardless of their income, which could present a financial burden. Third, low-income people may more reluctant to visit a dental clinic because of low public dental coverage. Choi et al. also reported that people in the low-income group were 4.46 times more likely to experience unmet dental care needs caused by economic burden in South Korea [34].

Expanding coverage for dental services is important for achieving universal health coverage. Palencia et al. reported that there were fewer inequalities in dental services in countries with high public dental coverage [35]. However, Tchicaya and Lorentz reported using multi-level analysis that the level of insurance coverage for basic dental care did not have a significant effect on the non-utilization of dental care, and they only accounted for small changes [36]. The impact of dental coverage and out-of-pocket costs on dental care utilization could be different across countries because of the different social contexts of their healthcare systems. Healthcare reform taking into consideration the social context of each country only can promote dental care in low-income households and alleviate inequality. In Korea, recent healthcare policy is mainly focused on expanding treatment coverage, such as dentures and implants for the elderly, light-curing dental composites for adolescents, and similar benefits. Healthcare reforms such as expanding the MAP and relieving insurance premiums and out-of-pocket costs for low-income people can also be considered and might strengthen access to dental care.

Although the NHIS dental scaling insurance policy relieved financial burdens and promoted dental scaling, it might not guarantee access to dental care. Access refers to the opportunity to use health services, and it has three dimensions: physical accessibility, financial affordability, and acceptability to patients [37, 38]. Listl et al. reported that the main reason for dental non-attendance in Europe in recent years was the public perception that regular dental treatment is “not necessary” or “not usual” [39]. In Korea, Kim et al. reported that 20.9% of those who reported experiencing unmet dental care needs cited an inability to leave work or school, and 14.5% of the respondents believed dental problems to be unimportant [40]. In Nova Scotia, even though all children were covered by a public dental insurance program, inequalities in dental visits and dental caries were observed to correspond to parental education levels [41]. Our results also showed differential levels of dental scaling usage according to age, residential area, education level, smoking status, and subjective oral health. To reduce inequalities in dental care, universal access to dental services should be considered in conjunction with universal coverage. For example, in Japan, the importance of oral health is taught in the schools to enhance acceptance of dental care [42]. Moreover, a law could be enacted requiring employers to provide employees extra vacation time for dental check-ups, although this has only been mandatory thus far for medical check-ups.

Targeting the healthcare system alone may have limited effects in reducing oral health inequalities. In Japan and Thailand, income-related inequalities in access to dental care services persisted even with the introduction of a universal dental insurance system [43, 44]. An individual’s socioeconomic status can affect oral health through various channels besides the healthcare system. Sanders et al. evaluated the role of individual dental behaviors in oral health inequalities and reported that individual dental behaviors and dental visits only partially accounted for the socioeconomic gradient in oral health inequalities [7]. There is also emerging evidence about the relationship between area-level income inequality and poor oral health. In the United States, state-level Gini coefficients were significantly associated with individual tooth loss even after adjustment for state- and individual-level confounders and potential mediators [5]. Material, behavioral/cultural, and psychosocial and other mechanisms have been posited to explain the association between individual- and area-level income inequality and worse oral health outcomes [15]. In these contexts, not only the healthcare system, but also the social structure and socio-environmental context account for the relationship between socioeconomic status and oral health outcomes. It also corresponds to Rose’s population strategy that targets the upstream cause of causes.

Proportionate universalism is suggested as a new approach to reduce health inequalities [45]. Under proportionate universalism, actions must be universal but with a scale and intensity that is proportionate to the level of disadvantage to reduce social gradient steepness in health [46]. A study from Glasgow, Scotland, reported that investment in housing-led renewal according to population need leads to modest reductions in area-based health inequalities after 5 years [47]. Additionally, a risk-based capitation model reduced socio-economic inequalities in dental caries among preschool children living in Sweden [48]. These results suggest that proportionate universalism can be an effective strategy to reduce oral health inequalities, but more evidence is necessary.

This research has some advantages. First, the CHS is a large, nationally representative survey that includes about 220,000 people annually, which enables us to estimate the effect of national policy accurately. Furthermore, the NHIS is a single-payer public healthcare system with universal coverage, meaning that everyone has the same benefits from the same insurer. The nationwide universal effect of health policies can be estimated based on the universal coverage.

This study has some limitations. Among these limitations, people living in hospitals, long-term medical facilities, and similar institutions were not included. These people could be more vulnerable and have worse oral health. Moreover, people with low health literacy could misunderstand the survey questions. However, as in the case of other national surveys, all questionnaires in the CHS are designed to maximize validity. Also, the CHS is based on a computer-assisted personal interviewing method, so the interviewer could help people with low health literacy. Finally, some recall bias is possible.

## Conclusion

This study examined the effect of the national dental scaling insurance policy on socioeconomic inequalities in dental scaling usage. Even though the policy improved overall dental scaling usage, socioeconomic inequalities in dental scaling were exacerbated. Because dental care access generally requires high individual agency, expanding dental coverage may have a limited effect in attenuating inequalities, inadvertently widening the gap. To reduce dental care inequalities, universal access with universal dental coverage should be considered. Furthermore, upstream and structural interventions targeting the socioeconomic context and new strategies such as proportionate universalism should be considered.

## Additional files

Additional file 1: **Figure S1.** Overview of the NHIS system. **Table S1.** Fee schedules of dental scaling at primary dental clinics in 2019. Explanation of the NHI and medical aid program, and reimbursement in each system / Fee schedule of dental scaling by cost-sharing in the pre- and post-policy periods. (DOCX 49 kb)

Additional file 2: **Figure S2.** Flow of inclusion criteria. Flow diagram of inclusion and exclusion of study population. (DOCX 30 kb)

Additional file 3: **Table S2.** Characteristics of people stratified by policy implementation using the Community Health Survey 2010–2016 (weighted %). Changes in demographics, socioeconomics variables, and health conditions between pre- and post-policy periods. (DOCX 21 kb)

Additional file 4: **Table S3.** Medians of each income category in pre- or post-policy periods using the Community Health Surveys 2010–12 and 2014–16 (weighted %). **Table S4.** Percentage and cut-off of per capita and equivalized monthly household income in pre- or post-policy periods using the Community Health Surveys 2010–12 and 2014–16 (weighted %). **Table S5.** Relative ratio and absolute differences of dental scaling non-user prevalence using equivalized household income levels in pre- or post-policy periods, Community Health Surveys 2010–12 and 2014–16 (weighted %). **Table S6.** Relative ratio and absolute differences of dental scaling non-user prevalence using per capita household income levels in pre- or post-policy periods, Community Health Surveys 2010–12 and 2014–16 (weighted %). Sensitivity analysis using equivalized and per-capita household income. (DOCX 32 kb)

## Data Availability

The data that support the findings of this study are available from KCDC [23], but restrictions apply to the availability of these data, which were used under license for the current study, and so are not publicly available. Data are however available from the authors upon reasonable request and with permission of KCDC.

## Abbreviations

CHS: Community Health Survey
KCDC: Korea Centers for Disease Control and Prevention
NHI: National Health Insurance
NHIS: National Health Insurance Service
SES: Socioeconomic status
