# Supplementary material for: Does the national dental scaling policy reduce inequalities in dental scaling usage? A population-based quasi-experimental study
Source: BMC Oral Health. 2019 Aug 14;19:185. doi: 10.1186/s12903-019-0881-7 (PMC6694626; doi:10.1186/s12903-019-0881-7)
Supplement: Supplementary file 1 — Figure S1. Overview of the NHIS system. Table S1. Fee schedules of dental scaling at primary dental clinics in 2019. Explanation of the NHI and medical aid program, and reimbursement in each system / Fee schedule of dental scaling by cost-sharing in the pre- and post-policy periods. (DOCX 49 kb) [file 12903_2019_881_MOESM1_ESM.docx]

The NHIS directs a two-segmented health insurance system, consisting of national health insurance (NHI) as social insurance and a medical aid program (MAP) as public assistance. NHI covers 97% of the population, and the premium is proportional to individual income. Physicians are reimbursed through a fee-for-services arrangement with out-of-pocket patient fees. The medical aid program, which offers almost the same benefits as the NHI, covers the low-income patients who make up the remaining 3% of the population. In the medical aid program, premiums are waived and out-of-pocket fees are minimized. Physicians are reimbursed at almost the same fee schedule as NHI patients.

**National Health Insurance (97.2%)***

**Medical aid**

**program (2.8% )***

**70%**

**30%**

**100%**

**by NHIS**

**By out of pocket**

**Covered services****

**NHIS system**

**For outpatient, at primary clinics**

**1000 KRW**

**per visit**

**100%**

**Non-covered services**

**National Health**

**Insurance**

**Medical aid**

**program**

***2018 The NHIS statistics**

****Almost the same benefits for the NHI and the medical aid program**

**Figure S1.**

Overview of the NHIS system in Korea

High-level cost-sharing is more dramatically shown in dental care. In Korea, the proportion of out-of-pocket fees in the current dental expenditure was approximately 84% in 2012. Covered dental care services, such as dental examination, extraction, amalgam restoration, endodontic treatment, etc. generally require 30% of cost-sharing in primary dental clinics, whereas dental clinics in hospitals require 40%. In case of non-covered dental care services such as dental prosthetics for those under 65, patients should pay the total cost. In these contexts, the NHIS is expanding benefits to reduce these financial burdens.

| **Table S1.** Fee schedules of dental scaling at primary dental clinics in pre- and post-policy periods (KRW). | | | |
| --- | --- | --- | --- |
| Reimbursement | Pre-policy period | Post-policy period (2019) | |
|  |  | National Health Insurance | Medical aid program |
| By out-of-pocket | Depends on each clinic, but totally patient's burden | 11,000 | 1,000* |
| By NHIS | X | 26,000 | 34,700 |
| Total fee | Depends on each clinic, but totally patient's burden | 37,000 | 35,700 |
| *per visit | | | |
