# Supplementary material for: Does the national dental scaling policy reduce inequalities in dental scaling usage? A population-based quasi-experimental study
Source: BMC Oral Health. 2019 Aug 14;19:185. doi: 10.1186/s12903-019-0881-7 (PMC6694626; doi:10.1186/s12903-019-0881-7)
Supplement: Supplementary file 2 — Figure S2. Flow of inclusion criteria. Flow diagram of inclusion and exclusion of study population. (DOCX 30 kb) [file 12903_2019_881_MOESM2_ESM.docx]

Community Health Survey 2010-2016 Initial population

N=1 601 879

Age under 20yr

N=17 971

People aged 20+yr

N=1 583 908

Without valid information on dental scaling

N=1167

People with valid dental scaling information

N=1 582 741

Without valid information on household income

N=65 644

N=1 517 097

Pre policy N=628 572

2013 N=218 261

Post policy N=670 264

**Figure S2**

Flow of inclusion criteria
