# Supplementary material for: Does the national dental scaling policy reduce inequalities in dental scaling usage? A population-based quasi-experimental study
Source: BMC Oral Health. 2019 Aug 14;19:185. doi: 10.1186/s12903-019-0881-7 (PMC6694626; doi:10.1186/s12903-019-0881-7)
Supplement: Supplementary file 3 — Table S2. Characteristics of people stratified by policy implementation using the Community Health Survey 2010–2016 (weighted %). Changes in demographics, socioeconomics variables, and health conditions between pre- and post-policy periods. (DOCX 21 kb) [file 12903_2019_881_MOESM3_ESM.docx]

| **Table S2.** Characteristics of people stratified by policy implementation using the Community Health Survey 2010–2016 (weighted %). | | | | | | | |
| --- | --- | --- | --- | --- | --- | --- | --- |
|  | | | | | Pre-policy (2010-2012) |  | Post-policy (2014-2016) |
| Scaling history in the past year | | | | Yes | 30.5 |  | 40.1 |
|  | | | | No | 69.5 |  | 59.9 |
|  | | |  | |  |  |  |
| Age | | | 20-34 | | 27.2 |  | 25.0 |
|  | | | 35-44 | | 23.1 |  | 20.7 |
|  | | | 45-54 | | 21.5 |  | 21.4 |
|  | | | 55-64 | | 13.9 |  | 16.3 |
|  | | | 65 and older | | 14.3 |  | 16.6 |
|  | | |  | |  |  |  |
| Sex | | | Male | | 49.7 |  | 49.5 |
|  | | | Female | | 50.3 |  | 50.5 |
|  | | |  | |  |  |  |
| Monthly household | | | < 1 | | 10.9 |  | 12.1 |
| income (10000 KRW) | | | 1 - 3 | | 35.6 |  | 32.9 |
|  | | | 3 - 5 | | 31.0 |  | 32.6 |
|  | | | ≥ 5 | | 22.4 |  | 22.3 |
|  | | |  | |  |  |  |
| Residence area | | | Urban | | 80.6 |  | 81.4 |
|  | | | Rural | | 19.4 |  | 18.6 |
|  | | |  | |  |  |  |
| Education | Under 6 years | | | | 14.6 |  | 13.1 |
|  | 6-9 years | | | | 9.4 |  | 8.7 |
|  | 10-12 years | | | | 31.3 |  | 30.1 |
|  | More than 12 years | | | | 44.5 |  | 48.0 |
|  | Missing | | | | 0.1 |  | 0.1 |
|  | | |  | |  |  |  |
| Insurance status | | MAP | | | 2.7 |  | 2.6 |
|  | | Former MAP | | | 0.7 |  | 0.7 |
|  | | NHI | | | 96.6 |  | 96.7 |
|  | |  | | |  |  |  |
| Current smoking | | | Current smoker | | 24.3 |  | 21.7 |
|  | | | Former smoker | | 15.5 |  | 17.3 |
|  | | | Non-smoker | | 60.1 |  | 61.0 |
|  | | |  | |  |  |  |
| Subjective oral health | | | Very good | | 4.3 |  | 3.8 |
|  | | | Good | | 24.5 |  | 21.3 |
|  | | | Moderate | | 36.5 |  | 41.7 |
|  | | | Bad | | 28.7 |  | 27.1 |
|  | | | Very bad | | 6.0 |  | 6.1 |
