# Supplementary material for: Does the national dental scaling policy reduce inequalities in dental scaling usage? A population-based quasi-experimental study
Source: BMC Oral Health. 2019 Aug 14;19:185. doi: 10.1186/s12903-019-0881-7 (PMC6694626; doi:10.1186/s12903-019-0881-7)
Supplement: Supplementary file 4 — Table S3. Medians of each income category in pre- or post-policy periods using the Community Health Surveys 2010–12 and 2014–16 (weighted %). Table S4. Percentage and cut-off of per capita and equivalized monthly household income in pre- or post-policy periods using the Community Health Surveys 2010–12 and 2014–16 (weighted %). Table S5. Relative ratio and absolute differences of dental scaling non-user prevalence using equivalized household income levels in pre- or post-policy periods, Community Health Surveys 2010–12 and 2014–16 (weighted %). Table S6. Relative ratio and absolute differences of dental scaling non-user prevalence using per capita household income levels in pre- or post-policy periods, Community Health Surveys 2010–12 and 2014–16 (weighted %). Sensitivity analysis using equivalized and per-capita household income. (DOCX 32 kb) [file 12903_2019_881_MOESM4_ESM.docx]

| **Table S3**. Medians of each income category in pre- or post-policy periods using the Community Health Survey 2010-12 and 2014-16 (weighted %) | | | | | | |
| --- | --- | --- | --- | --- | --- | --- |
| Monthly household income (million KRW) |  | Pre-policy | |  | Post-policy | |
|  |  | % | Median |  | % | Median |
| Up to 0.5 |  | 4.0 | 0.25 |  | 4.3 | 0.25 |
| 0.5 ≤ < 1.0 |  | 6.9 | 0.75 |  | 7.8 | 0.75 |
| 1.0 ≤ < 2.0 |  | 15.7 | 1.50 |  | 13.8 | 1.50 |
| 2.0 ≤ < 3.0 |  | 19.9 | 2.50 |  | 19.2 | 2.50 |
| 3.0 ≤ < 4.0 |  | 18.7 | 3.50 |  | 18.8 | 3.50 |
| 4.0 ≤ < 5.0 |  | 12.3 | 4.50 |  | 13.8 | 4.50 |
| 5.0 ≤ < 6.0 |  | 11.5 | 5.50 |  | 8.8 | 5.50 |
| More than 6.0 |  | 10.9 | 7.15* |  | 13.5 | 7.71* |
| * Median value was calculated based on Pareto’s curve | | | | | | |

| **Table S4**. Percentage and cut-off of per capita and equivalized monthly household income in pre- or post-policy periods using the Community Health Survey 2010-12 and 2014-16 (weighted %) | | | | | | | |
| --- | --- | --- | --- | --- | --- | --- | --- |
| Monthly household income (million KRW) | Pre-policy | | |  | Post-policy | | |
|  | % |  | Cut-off |  | % |  | Cut-off |
| Per capita |  |  |  |  |  |  |  |
| 1st | 19.8 |  | < 58.3 |  | 18.1 |  | < 62.5 |
| 2nd | 16.7 |  | < 83.3 |  | 20.8 |  | < 87.5 |
| 3rd | 21.5 |  | < 116.7 |  | 21.0 |  | < 125.0 |
| 4th | 22.0 |  | < 175.0 |  | 17.7 |  | < 175.0 |
| 5th | 20.0 |  |  |  | 22.4 |  |  |
| Equivalized |  |  |  |  |  |  |  |
| 1st | 19.4 |  | < 106.1 |  | 18.2 |  | < 106.1 |
| 2nd | 19.9 |  | < 150.0 |  | 20.3 |  | < 156.5 |
| 3rd | 17.9 |  | < 202.1 |  | 21.5 |  | < 207.9 |
| 4th | 20.2 |  | < 275.0 |  | 16.2 |  | < 275.0 |
| 5th | 22.6 |  |  |  | 23.8 |  |  |

| **Table S5**. Relative ratio and absolute differences of dental scaling non-users prevalence using equivalized household income levels in pre- or post-policy periods, Community Health Survey 2010-12 and 2014-16 (weighted %) | | | | | | | | | |
| --- | --- | --- | --- | --- | --- | --- | --- | --- | --- |
| **Equivalized**  **monthly household income (million KRW)** | Dental scaling  non-user prevalence (%) | | | | Relative ratio | | | Absolute difference % | |
|  | Pre- | Post- | Difference |  | Pre- | Post- |  | Pre- | Post- |
| Crude |  |  |  |  |  |  |  |  |  |
| 1st | 57.3 | 48.3 | -9.0 |  | 1 | 1 |  | 0 | 0 |
| 2nd | 66.0 | 54.9 | -11.1 |  | 1.15 | 1.13 |  | 8.7 | 6.6 |
| 3rd | 69.8 | 59.3 | -10.5 |  | 1.22 | 1.23 |  | 12.6 | 11.1 |
| 4th | 74.5 | 64.6 | -9.9 |  | 1.30 | 1.34 |  | 17.2 | 16.3 |
| 5th | 82.1 | 75.1 | -7.0 |  | 1.43 | 1.56 |  | 24.8 | 26.9 |
| *Adjusted |  |  |  |  |  |  |  |  |  |
| 1st | 61.4 | 51.6 | -9.8 |  | 1 | 1 |  | 0 | 0 |
| 2nd | 68.4 | 57.2 | -11.2 |  | 1.11 | 1.11 |  | 7.0 | 5.6 |
| 3rd | 70.7 | 60.5 | -10.2 |  | 1.15 | 1,17 |  | 9.3 | 8.9 |
| 4th | 73.0 | 63.2 | -9.8 |  | 1.19 | 1.22 |  | 11.6 | 11.6 |
| 5th | 74.2 | 67.1 | -7.0 |  | 1.21 | 1.30 |  | 12.7 | 15.5 |
| *A multivariable Poisson regression was used with adjusting age, sex, residence area, education years, current smoking, insurance status, and subjective oral health. | | | | | | | | | |

| **Table S6**. Relative ratio and absolute differences of dental scaling non-users prevalence using per capita household income levels in pre- or post-policy periods, Community Health Survey 2010-12 and 2014-16 (weighted %) | | | | | | | | | |
| --- | --- | --- | --- | --- | --- | --- | --- | --- | --- |
| **Per capita**  **monthly household income (million KRW)** | Dental scaling  non-user prevalence | | | | Relative ratio | | | Absolute difference % | |
|  | Pre- | Post- | Difference |  | Pre- | Post- |  | Pre- | Post- |
| Crude |  |  |  |  |  |  |  |  |  |
| 1st | 57.8 | 48.9 | -8.9 |  | 1 | 1 |  | 0 | 0 |
| 2nd | 66.0 | 55.5 | -10.5 |  | 1.14 | 1.13 |  | 8.1 | 6.6 |
| 3rd | 68.5 | 57.5 | -11.0 |  | 1.18 | 1.18 |  | 10.6 | 8.7 |
| 4th | 75.3 | 65.4 | -9.9 |  | 1.30 | 1.34 |  | 17.5 | 16.5 |
| 5th | 81.4 | 74.4 | -7.0 |  | 1.41 | 1.52 |  | 23.6 | 25.5 |
| *Adjusted |  |  |  |  |  |  |  |  |  |
| 1st | 61.7 | 52.1 | -9.6 |  | 1 | 1 |  | 0 | 0 |
| 2nd | 68.0 | 57.0 | -11.0 |  | 1.10 | 1.09 |  | 6.3 | 4.9 |
| 3rd | 70.6 | 60.0 | -10.6 |  | 1.14 | 1.15 |  | 8.9 | 7.9 |
| 4th | 72.8 | 63.4 | -9.4 |  | 1.18 | 1.22 |  | 11.1 | 11.3 |
| 5th | 74.1 | 66.9 | -7.2 |  | 1.20 | 1.28 |  | 12.4 | 14.8 |
| *A multivariable Poisson regression was used with adjusting age, sex, residence area, education years, current smoking, insurance status, and subjective oral health. | | | | | | | | | |
